# Burnout Among Italian Nurses: Organizational Correlates, Workload Burden, and Workforce Sustainability in a National Cross-Sectional Study

**DOI:** 10.3390/ijerph23070890

**Published:** 2026-07-10

**Authors:** Angelo Cianciulli, Giovanni Boccia, Marco Ferrara, Antonietta Pacifico, Roberta Manente, Michele Nappa, Domenico Fornino, Anna Di Gisi, Emanuela Nuccio, Fabrizio Liguori, Emanuela Santoro

**Affiliations:** 1 Department of Medicine, Surgery and Dentistry ‘’Scuola Medica Salernitana”, University of Salerno, 84081 Salerno, Italy; ancianciulli@unisa.it (A.C.); gboccia@unisa.it (G.B.); marcoferrara2708@gmail.com (M.F.); apacifico@unisa.it (A.P.); mnappa@unisa.it (M.N.); 2 Integrated Care Department of Health Hygiene and Evaluative Medicine, San Giovanni di Dio and Ruggi d’Aragona University Hospital, 84131 Salerno, Italy; 3 Hospital and Epidemiological Hygiene Unit, San Giovanni di Dio and Ruggi d’Aragona University Hospital, 84131 Salerno, Italy; 4 San Giovanni di Dio and Ruggi d’Aragona University Hospital, 84081 Salerno, Italy; manente392@gmail.com; 5 Medical Directorate, San Giovanni di Dio and Ruggi d’Aragona University Hospital, 84081 Salerno, Italy; domenico.fornino@sangiovannieruggi.it; 6 Department of Health, Health Professions Service, San Giovanni di Dio and Ruggi d’Aragona University Hospital, 84081 Salerno, Italy; anna.digisi@sangiovannieruggi.it; 7 Department of Health Services, Medical Directorate of Hospital Facilities, A.O.R.N. “San Giuseppe Moscati”, 83100 Avellino, Italy; emanuelanuccio1986@gmail.com; 8 Department of Medical, Movement Sciences and Wellbeing, University of Naples “Parthenope”, 80133 Naples, Italy; fabrizio.liguori91@gmail.com

**Keywords:** burnout, nurses, Copenhagen Burnout Inventory, job satisfaction, workload, Italy, occupational health

## Abstract

**Highlights:**

**Public health relevance—How does this work relate to a public health issue?**

- Burnout among nurses represents a major occupational health challenge associated with workforce instability, reduced care quality, and increased patient safety risks.
- Organizational factors such as excessive workload and low job satisfaction are modifiable contributors to burnout and are highly relevant for healthcare system sustainability.

**Public health significance—Why is this work of significance to public health?**

- This nationwide online survey provides updated post-pandemic evidence on burnout among Italian nurses using a validated and internationally comparable instrument.
- The findings reinforce the interpretation of burnout as a system-level healthcare issue linked to workforce retention, organizational resilience, and quality of care.

**Public health implications—What are the key implications or messages for practitioners, policy makers and/or researchers in public health?**

- Healthcare organizations should integrate burnout monitoring into workforce governance strategies alongside staffing adequacy, turnover, and patient safety indicators.
- Policies aimed at improving working conditions, leadership support, and professional satisfaction may contribute to lower burnout, improved nurse retention, and safer healthcare systems.

**Abstract:**

Background: Nurse burnout is a major occupational health problem with implications for workforce retention, quality of care, and patient safety. Contemporary evidence consistently links burnout to increased missed care, adverse events, and reduced safety climate, underscoring the need for updated national data and identification of modifiable organizational factors. Objective: To describe burnout levels among Italian nurses using the Copenhagen Burnout Inventory (CBI) and to examine associations between burnout, job satisfaction, weekly working hours, and key sociodemographic and professional characteristics. Methods: We conducted a national, observational cross-sectional online survey (Google Forms) between April and May 2026. Convenience and snowball sampling were used via professional social media channels and nursing-related institutional pages. Burnout was measured using the Copenhagen Burnout Inventory (CBI; 19 items covering personal, work-related, and patient-related burnout). Responses were converted to standardized 0–100 scores (higher scores = higher burnout); total burnout was computed as the mean of the three subscales. Job satisfaction was assessed on a 1–5 Likert scale. Group comparisons used Mann–Whitney U and Kruskal–Wallis tests; associations used Spearman’s rho; multivariable linear regression was fitted to examine factors independently associated with burnout. Results: The sample included 557 nurses; 84.6% were women, 37.0% were aged <30 years, and participants covered all Italian macro-areas (North 52.4%, Centre 21.5%, South 12.4%, Islands 13.6%). Mean burnout scores (0–100) were: total 50.80 ± 18.87, personal 60.11 ± 19.53, work-related 53.30 ± 22.25, patient-related 38.99 ± 22.77. Using conventional cut-offs (<33 low; 33–66 moderate; >66 high), 19.4% had low, 59.8% moderate, and 20.8% high burnout. Total burnout correlated positively with weekly working hours (ρ = 0.180, *p* < 0.001) and negatively with job satisfaction (ρ = −0.620, *p* < 0.001). In multivariable models, higher job satisfaction remained the strongest independent factor associated with lower burnout, while working >40 h/week remained independently associated with higher burnout. Conclusions: Burnout among Italian nurses was prevalent at moderate-to-high levels. Job satisfaction emerged as the main organizational factor associated with burnout, while higher workload was also significantly associated with greater burnout levels. These findings highlight the importance of organizational strategies addressing working conditions to support nurse well-being and patient safety.

## 1. Introduction

Burnout among nurses has emerged as a persistent and system-level challenge, increasingly framed as both a workforce sustainability issue and a patient safety concern. Recent high-quality synthesis evidence demonstrates that nurse burnout is significantly associated with poorer safety climate, increased missed care, lower patient satisfaction, and worse nurse-assessed quality of care, confirming that burnout is not merely an individual psychological phenomenon but a key determinant of healthcare system performance [1].

Post-pandemic healthcare systems are facing unprecedented pressures, including chronic staffing shortages, increased patient complexity, skill-mix imbalances, and growing administrative demands [2,3,4]. These pressures have accelerated workforce instability and intentions to leave, further amplifying workload and compromising team functioning [5,6]. In parallel, evidence indicates that extended work hours, mandatory overtime and rotating shifts are consistently associated with higher burnout and lower job satisfaction among nurses [7,8]. Importantly, modifiable organizational drivers—such as workload, staffing adequacy, leadership quality, perceived organizational support, and work–life balance—have emerged as central determinants of burnout, while meaningful work, supportive management, and professional autonomy act as protective factors [2,3,4,9,10,11,12,13,14].

Burnout is also strongly intertwined with workforce instability. Large European multicentre studies show that emotional exhaustion, dissatisfaction with professional development, and perceived underutilization of competencies significantly increase nurses’ intentions to leave both their organizations and the profession [5,15,16]. In the context of the ongoing nursing shortage in Italy and other European countries, understanding the organizational correlates of burnout is therefore essential for evidence-informed workforce and retention policies.

The choice of measurement instrument is critical. While Maslach’s conceptualization of burnout remains influential, contemporary occupational health research increasingly favors exhaustion-centered operationalizations that allow for pragmatic organizational interpretation. The Copenhagen Burnout Inventory (CBI) is widely adopted internationally because it distinguishes between personal, work-related, and patient-related burnout dimensions, facilitating more targeted prevention strategies [6]. In healthcare settings, the patient-related dimension is commonly operationalized as patient-related burnout. In Italy, the use of a culturally validated version is essential for psychometric accuracy and comparability [7,8].

Despite a growing literature on nurse well-being in Europe, large-sample national data from Italy integrating burnout, job satisfaction, workload, and professional characteristics remain limited, particularly in the post-pandemic period. Moreover, evidence from high-intensity clinical settings, such as critical care, shows persistently elevated levels of psychological distress and burnout among nurses, reinforcing the urgency of identifying actionable organizational levers [9].

Finally, interpretation of observational and secondary evidence requires methodological rigor. Recent meta-epidemiological research has shown that even systematic reviews frequently apply risk-of-bias tools incorrectly, underlining the importance of transparent methods and cautious inference when translating evidence into practice [17]. Within this framework, survey-based occupational health studies must demonstrate strong measurement validity and analytical transparency.

The present research group has extensive experience in questionnaire validation and outcomes-oriented clinical research in Italy, including the validation of patient-reported outcome measures in post-COVID conditions [18] and evidence-based evaluations in wound care and perioperative nutrition [19,20], supporting the methodological reliability of the present survey. In the Italian post-pandemic context, organizational pressures have intensified, making the analysis of modifiable factors, such as workload and job satisfaction, crucial to preserve nursing workforce sustainability and care quality. Within this evolving organizational landscape, identifying modifiable occupational factors associated with burnout is increasingly relevant for workforce governance, retention strategies, and patient safety improvement initiatives.

The present study was conceptually informed by the Job Demands–Resources (JD-R) model, which proposes that excessive job demands contribute to occupational strain and burnout, whereas organizational resources promote work engagement and psychological well-being. Within this framework, weekly working hours can be conceptualized as a proxy for job demands, whereas overall job satisfaction may reflect the availability of organizational resources and supportive working conditions. This theoretical perspective therefore guided both the selection of study variables and the interpretation of the observed associations.

Therefore, the aims of this study were to (i) quantify burnout levels among Italian nurses using the CBI; (ii) examine associations between burnout, job satisfaction, weekly working hours, and professional characteristics; and (iii) explore organizational and professional factors associated with burnout to inform organizational and managerial interventions.

## 2. Materials and Methods

### 2.1. Study Design and Setting

We conducted a national, observational, cross-sectional survey of nurses working in Italy. Data were collected between April and May 2026 through an anonymous online questionnaire administered via Google Forms (Google LLC, Mountain View, CA, USA). This study was conducted and reported in accordance with the Strengthening the Reporting of Observational Studies in Epidemiology (STROBE) guidelines for cross-sectional studies [21].

### 2.2. Recruitment and Participants

A non-probabilistic convenience sampling approach was used, complemented by snowball dissemination via nursing-related social media channels and professional pages. Inclusion criteria were: registered nurse, age ≥ 18 years, currently working in Italy, and at least 6 months of continuous work experience. Nursing students, retirees, non-nursing professionals, and incomplete or incoherent questionnaires were excluded. A total of 557 complete responses were included in the analysis. Because recruitment relied on convenience and snowball sampling through online dissemination, the study was not designed to obtain a statistically representative sample of the Italian nursing workforce. Participants were encouraged to share the survey invitation with eligible nursing colleagues working in different healthcare settings across Italy, thereby facilitating snowball dissemination. Rather, the objective was to obtain a geographically heterogeneous nationwide sample to explore associations between burnout and selected occupational characteristics.

The survey link was through professional nursing social media platforms (Facebook, Instagram, LinkedIn, and WhatsApp), Provincial Nursing Boards (OPIs), professional mailing lists, and online nursing communities. Participation was entirely voluntary, and no financial or non-financial incentives were offered. Because the survey was openly disseminated through multiple professional online platforms and networks, no predefined sampling frame was available and the total number of individuals who viewed the survey invitation could not be accurately determined. Consequently, a response rate could not be reliably calculated. This limitation is inherent to open online recruitment strategies and has been considered when interpreting the findings. This limitation is inherent to open online recruitment strategies relying on social media dissemination and has been acknowledged in the interpretation of the findings.

Before accessing the questionnaire, all participants were presented with an electronic information page describing the aims of the study, the voluntary nature of participation, data confidentiality, and anonymity. Electronic informed consent was obtained before participants could proceed to the questionnaire. Participants were free to discontinue the survey at any time before submission without providing any justification.

### 2.3. Ethics and Data Protection

The study was reviewed by the Ethics Committee Campania 3, which issued a formally acknowledged the study of the anonymous online survey, as reported in meeting record 07/26 of 1 April 2026 and transmitted with Prot. No. 00017397 on 9 April 2026. Given the anonymous, observational and non-interventional nature of the survey, the study was conducted in accordance with the principles of the Declaration of Helsinki and applicable GDPR requirements.

### 2.4. Data Collection Instruments

#### 2.4.1. Copenhagen Burnout Inventory (CBI)

Burnout was assessed using the CBI (19 items), comprising personal burnout (6 items), work-related burnout (7 items), and patient-related burnout (6 items) [6,7]. The Italian validated version was used; the validation materials were requested from Prof. Lorenzo Avanzi on 10 November 2025 [8].

Responses were scored on a 5-point Likert scale and converted to standardized 0–100 values (0, 25, 50, 75, 100), including reverse scoring for the energy item. Subscale scores were calculated as the means of their respective items, and total burnout as the mean of the three subscales.

Because all questionnaire items were mandatory within Google Forms, incomplete questionnaires could not be submitted. Prior to statistical analysis, the dataset was additionally reviewed to identify any evident inconsistencies or anomalous records; no responses required exclusion after this verification. Responses were reviewed for completeness before inclusion in the analytical dataset. Google Forms settings were configured to allow only one response per participant, thereby preventing duplicate questionnaire submissions.

#### 2.4.2. Job Satisfaction

Job satisfaction was measured using a single 5-point Likert item (1 = not satisfied at all; 5 = very satisfied).

Overall job satisfaction was assessed using a single global five-point Likert item ranging from 1 (“very dissatisfied”) to 5 (“very satisfied”). A single-item measure was selected to minimize respondent burden and maximize participation in this nationwide online survey. Single-item measures of overall job satisfaction have been widely used in occupational health research and have demonstrated acceptable validity when the objective is to capture respondents’ overall perception of their work rather than its individual dimensions. However, unlike multidimensional instruments such as the Minnesota Satisfaction Questionnaire (MSQ), the Job Satisfaction Survey (JSS), or the McCloskey/Mueller Satisfaction Scale, this approach does not allow identification of the specific organizational components underlying job satisfaction. The item was specifically developed to capture respondents’ overall perception of job satisfaction within the context of the present nationwide survey and was not intended to replace multidimensional job satisfaction instruments.

#### 2.4.3. Other Variables

Sociodemographic and professional variables included gender, age group, geographic area, educational level, years of professional experience, contract type, healthcare setting, department macro-area, shift pattern, and weekly working hours. Free-text occupational variables were reviewed and harmonized into analytically meaningful categories before descriptive analysis.

#### 2.4.4. Statistical Analysis

Descriptive statistics are presented as frequencies and percentages for categorical variables and as means ± standard deviations or medians (interquartile ranges) for continuous variables, as appropriate. Cases with missing data on any of the study variables were handled using listwise deletion. Group comparisons were conducted using Mann–Whitney U tests and Kruskal–Wallis tests. Distributional assumptions were assessed using Shapiro–Wilk tests and visual inspection of histograms and Q–Q plots. Given the ordinal nature of several predictors and the distributional characteristics of burnout scores, non-parametric tests were used for bivariate comparisons. Associations between continuous variables were examined using Spearman’s rank correlation coefficients. Independent variables were selected a priori based on the job demands–resources model and previous empirical evidence linking workload and job satisfaction to burnout among nurses. A multivariable linear regression model was fitted with total burnout score as the dependent variable and job satisfaction, working more than 40 h per week, gender, geographic area, age group, and years of professional experience as independent variables. Statistical significance was set at *p* < 0.05. Assumptions of linearity, homoscedasticity, and independence of residuals were verified through residual analysis. Multicollinearity was excluded by assessing Variance Inflation Factors (VIF), below 2 were considered indicative of negligible multicollinearity. All statistical analyses were performed using R software (version 4.3.2; R Foundation for Statistical Computing, Vienna, Austria) [22]. Effect sizes and confidence intervals were interpreted alongside *p*-values to improve the clinical and organizational interpretability of the findings.

## 3. Results

### 3.1. Sample Characteristics

The final sample consisted of 557 nurses. Most participants were women (n = 471, 84.6%), while 85 were men (15.3%) and one participant preferred not to disclose gender. The largest age groups were <30 years (n = 206, 37.0%) and 30–39 years (n = 191, 34.3%), followed by 50 years or older (n = 84, 15.1%) and 40–49 years (n = 76, 13.6%).

Participants were distributed across all Italian macro-areas, with the largest proportion working in Northern Italy (n = 292, 52.4%), followed by Central Italy (n = 120, 21.5%), Islands (n = 76, 13.6%), and Southern Italy (n = 69, 12.4%). Most nurses held a bachelor’s degree (n = 353, 63.4%), while 129 participants reported a postgraduate master’s degree (23.2%) and 48 a master’s degree in nursing sciences (8.6%).

Regarding professional characteristics, 210 nurses had less than 5 years of professional experience (37.7%), 163 had 5–10 years (29.3%), 85 had 11–20 years (15.3%), 52 had 21–30 years (9.3%), and 47 had more than 30 years of experience (8.4%). Most participants were employed with permanent contracts (n = 470, 84.4%). Weekly working hours were mainly 30–36 h (n = 253, 45.4%) or 37–40 h (n = 246, 44.2%), while 39 nurses reported working more than 40 h per week (7.0%).

Job satisfaction was distributed as follows: 54 nurses reported very low satisfaction (9.7%), 102 low satisfaction (18.3%), 212 moderate satisfaction (38.1%), 154 high satisfaction (27.6%), and 35 very high satisfaction (6.3%). Sociodemographic and professional characteristics are shown in Table 1.

### 3.2. Burnout Levels

Mean total burnout (CBI total score) was 50.80 ± 18.87, indicating a moderate overall level of burnout. Subscale scores were highest for personal burnout (60.11 ± 19.53), followed by work-related burnout (53.30 ± 22.25) and patient-related burnout (38.99 ± 22.77).

Using conventional CBI cut-offs (<33 = low; 33–66 = moderate; >66 = high burnout), 19.4% of nurses had low, 59.8% moderate, and 20.8% high total burnout. Descriptive statistics for the CBI subscales and total score are reported in Table 2.

Based on conventional CBI cut-offs, 108 nurses (19.4%) showed low burnout, 333 (59.8%) moderate burnout, and 116 (20.8%) high burnout. Therefore, approximately four out of five nurses reported moderate-to-high burnout levels. From an organizational perspective, the observed prevalence of moderate-to-high burnout is clinically relevant. Contemporary evidence consistently links elevated burnout levels with increased missed nursing care, reduced patient safety culture, higher turnover intention, and lower workforce retention. Therefore, the present findings should be interpreted not only as indicators of individual distress, but also as markers of organizational vulnerability within healthcare systems. Distribution of burnout severity categories is presented in Table 3.

### 3.3. Correlations Between Burnout, Job Satisfaction and Working Hours

Total burnout showed a weak but statistically significant positive correlation with weekly working hours category (Spearman’s ρ = 0.180, *p* < 0.001), indicating that higher workload was associated with higher burnout. Conversely, job satisfaction showed a strong inverse correlation with total burnout (ρ = −0.620, *p* < 0.001), suggesting that nurses reporting higher job satisfaction had substantially lower burnout scores.

The CBI subscales were all significantly intercorrelated. The strongest association was observed between personal and work-related burnout (ρ = 0.861, *p* < 0.001), indicating a substantial overlap between general exhaustion and work-related exhaustion. Patient-related burnout showed moderate correlations with both personal burnout (ρ = 0.521, *p* < 0.001) and work-related burnout (ρ = 0.569, *p* < 0.001). The stronger correlation observed between personal and work-related burnout, compared with patient-related burnout, suggests that organizational and workload-related dimensions may contribute more substantially to burnout than patient interaction itself within the present nursing sample.

**Figure 1:**
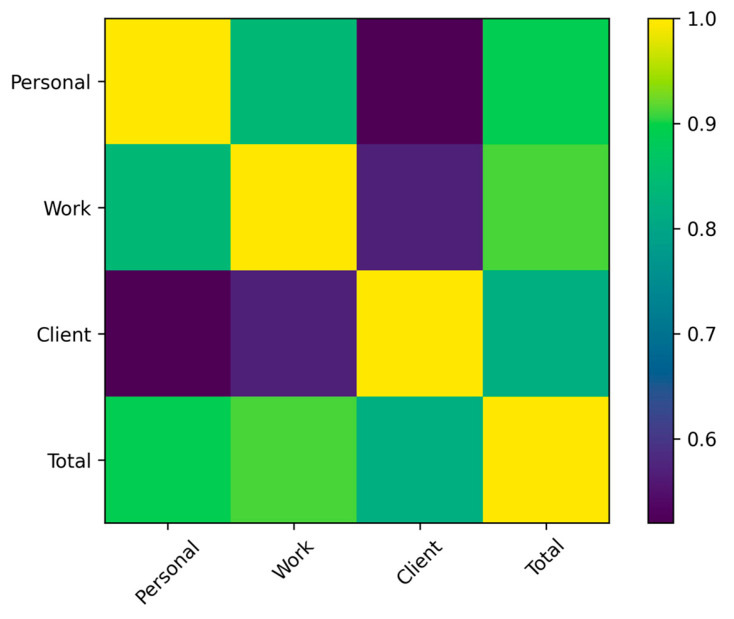
Correlation Heatmap of Burnout Dimensions, Job Satisfaction, and Weekly Working Hours.

Bivariate associations between burnout dimensions and job satisfaction are presented in Table 4, while the correlation structure among CBI subscales is illustrated in Figure 1.

### 3.4. Reliability Analysis

Internal consistency was good to excellent across the CBI dimensions. Cronbach’s alpha was 0.878 for personal burnout, 0.904 for work-related burnout, 0.883 for patient-related burnout, and 0.941 for the total CBI scale. Internal consistency coefficients for the CBI dimensions are reported in Table 5.

### 3.5. Multivariable Analysis of Factors Associated with Burnout

In multivariable linear regression analysis, job satisfaction showed the strongest independent association with lower burnout. Specifically, each one-point increase in job satisfaction was associated with an approximately 11.8-point reduction in total burnout scores (β = −11.80, *p* < 0.001).

Working more than 40 h per week also remained independently associated with significantly higher burnout after adjustment for all covariates (β = 7.19, *p* = 0.007).

Gender and geographic area were not independently associated with burnout in the adjusted model. Among age categories, nurses aged 50 years or older showed significantly lower burnout scores compared with the reference category. In contrast, nurses with longer professional experience (>20 years) showed significantly higher burnout scores.

The results of the multivariable linear regression model associated with total burnout are presented in Table 6. The final model explained a substantial proportion of variance in burnout scores (Adjusted R^2^ = 0.398). Geographic area was not independently associated with total burnout in the adjusted model. No evidence of problematic multicollinearity was observed among predictors. Residual diagnostics showed no major deviations from linearity or homoscedasticity assumptions. Variance Inflation Factors were all below commonly accepted thresholds, supporting the stability of the regression estimates. Overall, the regression findings reinforce the interpretation of burnout as a multidimensional occupational phenomenon strongly influenced by modifiable organizational conditions, particularly perceived job satisfaction and workload burden.

From an organizational perspective, the strong inverse association observed between job satisfaction and burnout suggests that perceived workplace satisfaction represents an important organizational correlate of burnout. However, given the cross-sectional design, these findings should not be interpreted as evidence of a causal relationship.

### 3.6. Burnout Across Professional Experience

**Figure 2:**
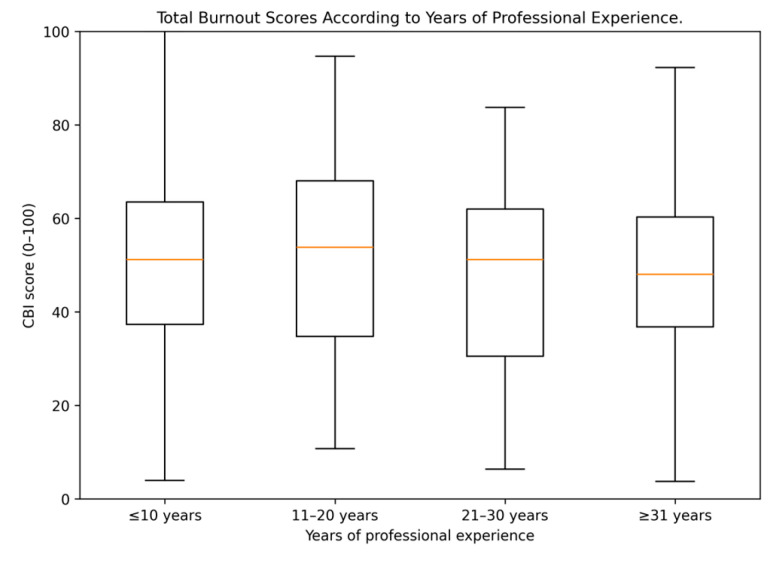
Total Burnout Scores According to Years of Professional Experience.

**Figure 3:**
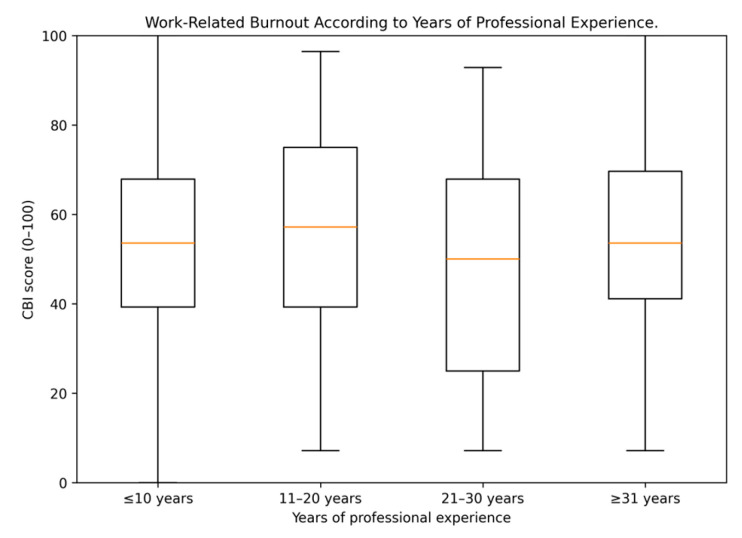
Work-Related Burnout According to Years of Professional Experience.

**Figure 4:**
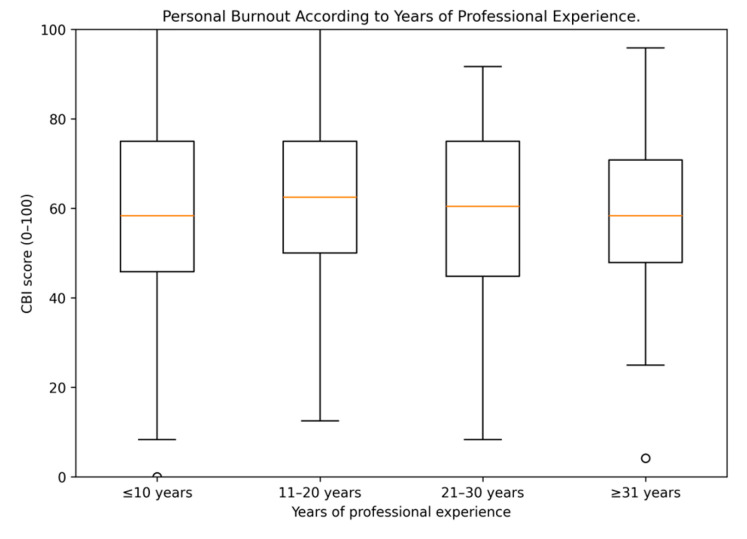
Personal Burnout According to Years of Professional Experience.

**Figure 5:**
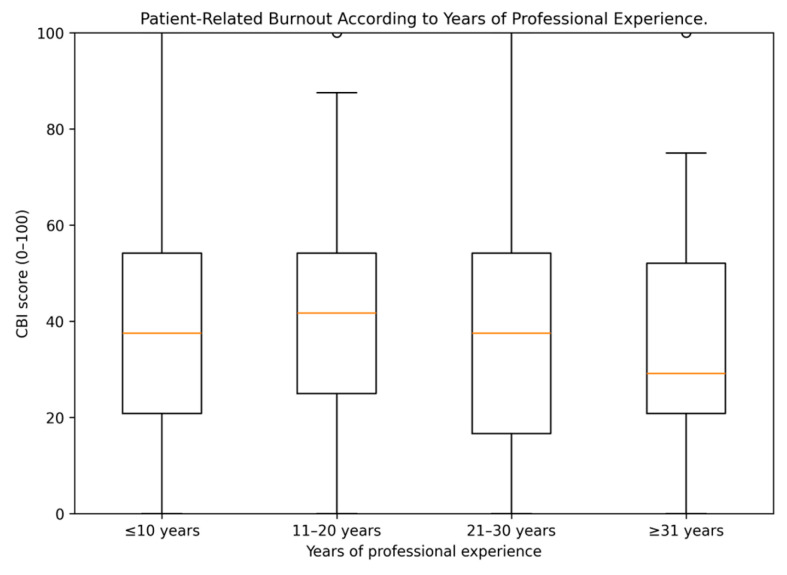
Patient-Related Burnout According to Years of Professional Experience.

Exploratory graphical analyses suggested higher burnout levels among early-career and mid-career nurses, whereas lower levels were generally observed among the most experienced professionals. This pattern was consistent across total, personal, work-related, and patient-related burnout dimensions (Figure 2, Figure 3, Figure 4 and Figure 5).

## 4. Discussion

The present national survey shows that burnout among Italian nurses is highly prevalent, with approximately 80% of participants reporting moderate-to-high total burnout levels. The highest mean score was observed for personal burnout, followed by work-related burnout, whereas patient-related burnout was comparatively lower. This pattern suggests that exhaustion in this sample was mainly associated with general and organizational dimensions of work rather than with patient interaction per se [23,24]. This interpretation is coherent with contemporary occupational health models emphasizing workload pressure, organizational climate, and workforce instability as central mechanisms underlying burnout among healthcare professionals.

The very strong inverse association between job satisfaction and burnout observed in our sample mirrors a growing international literature demonstrating that job satisfaction is not merely associated with well-being but appears to represent an important organizational correlate of burnout, turnover intention, and quiet quitting [25,26,27,28]. Recent longitudinal evidence indicates that nurses reporting low job satisfaction are substantially more likely to develop emotional exhaustion and to consider leaving their organization within 12–24 months, even after adjustment for age, seniority, and work setting [25,26]. These findings support the interpretation of job satisfaction as a potential indicator of organizational health rather than solely an individual characteristic, although causal relationships cannot be inferred from the present cross-sectional study.

Workload, operationalized in our study as weekly working hours, remained independently associated with higher burnout. From a workforce sustainability perspective, these findings are particularly concerning because chronic organizational overload may progressively reduce professional engagement, increase presenteeism, and accelerate workforce attrition. In healthcare systems already affected by nursing shortages, previous longitudinal and observational studies suggest that sustained exposure to burnout-related organizational stressors may compromise workforce stability and long-term care quality. The present findings are consistent with this broader body of evidence but do not directly demonstrate these outcomes. This is consistent with contemporary research showing that extended working hours, mandatory overtime, and unpredictable scheduling are associated with both burnout and reduced safety behaviours among nurses [27,29,30]. Importantly, recent post-pandemic analyses have demonstrated that workload pressure is also a key driver of quiet quitting, presenteeism, and disengagement, phenomena that further threaten workforce sustainability and care quality [28,30,31].

Beyond workload and satisfaction, psychological safety has been increasingly recognized as a critical organizational resource buffering burnout. Scoping and systematic reviews published in the last three years indicate that nurses working in environments characterized by open communication, supportive leadership, and non-punitive error reporting experience significantly lower burnout and higher professional engagement [32,33,34]. Although psychological safety was not directly measured in the present survey, the observed association between job satisfaction and burnout is consistent with previous literature suggesting that psychological safety may represent one of several organizational mechanisms underlying these relationships in healthcare settings.

Although psychological safety was not directly measured in the present study, previous empirical research consistently demonstrates that psychologically safe work environments are associated with higher job satisfaction, greater work engagement, and lower burnout. Therefore, the interpretation proposed here should be considered exploratory and informed by the broader literature rather than directly supported by the present data.

Interestingly, nurses aged 50 years or older showed lower burnout scores in the adjusted analysis, whereas very long professional experience was associated with higher burnout. This may reflect a complex interaction between professional adaptation, resilience, cumulative occupational burden, and career-stage organizational pressures.

The organizational relevance of burnout is further underscored by its well-documented links with patient safety and quality of care. High-quality meta-analyses and multicentre studies have shown that higher nurse burnout is associated with increased missed care, medication errors, poorer safety culture, and lower patient satisfaction [1,21,35,36,37]. Thus, the high prevalence of moderate-to-high burnout observed in this study should be interpreted not only as a workforce health signal, but also as a potential early warning indicator for care quality deterioration.

Burnout is also tightly connected to workforce stability. Recent European and international evidence demonstrates that emotional exhaustion mediates the relationship between organizational stressors and turnover intention, absenteeism, and early exit from the profession [26,29,38,39]. In this light, the independent associations of job satisfaction and workload identified in our multivariable models point to clear organizational levers that could be targeted by nurse managers and health system leaders. These findings also have direct implications for healthcare policy and organizational governance. Monitoring burnout indicators alongside staffing adequacy, turnover rates, and safety culture metrics could support more proactive workforce management strategies and earlier identification of organizational distress. These findings are particularly relevant in the context of ongoing nursing shortages across Europe, where maintaining workforce sustainability has become a strategic priority for healthcare systems.

To facilitate translation of the present findings into organizational and managerial practice, the main results and their potential implications for healthcare systems are summarized in Table 7.

These organizational implications reinforce the interpretation of burnout as a healthcare system issue requiring proactive workforce governance rather than exclusively individual-level coping interventions.

Methodologically, this study benefits from the use of a validated and internationally comparable burnout instrument and from transparent analytic procedures. This is particularly relevant in light of recent meta-epidemiological findings showing that even systematic reviews often misapply risk-of-bias tools and overestimate the certainty of evidence [17]. By providing detailed scoring procedures, robust descriptive statistics, and multivariable modelling, the present study contributes reproducible and policy-relevant evidence to the field. The excellent internal consistency observed across the CBI dimensions further supports the reliability of the instrument within the present nursing sample. Moreover, the relatively large national sample and the inclusion of nurses from all Italian macro-areas improve the external relevance of the findings and provide a broader overview of burnout patterns within the contemporary Italian nursing workforce. Although leadership quality and peer support were not directly assessed in the present study, previous research consistently identifies these organizational factors as important determinants of both job satisfaction and burnout. Therefore, the organizational strategies discussed here should be interpreted as evidence-informed implications derived from the broader literature rather than direct findings of the present study.

Nevertheless, several limitations should be acknowledged. First, the cross-sectional design precludes causal inference, and the observed associations should therefore be interpreted cautiously. Second, the convenience and snowball sampling strategy may have introduced self-selection bias. Nurses experiencing higher levels of occupational stress or burnout may have been more motivated to participate in the survey than those with lower levels of burnout. Consequently, the prevalence estimates reported in this study should be interpreted cautiously and should not be considered representative of the entire Italian nursing workforce. Instead, the findings should be viewed as evidence derived from a geographically heterogeneous nationwide online survey, providing valuable insights into associations between burnout and occupational characteristics rather than population-level prevalence estimates. Third, all variables were self-reported and may therefore be influenced by recall bias or subjective perception. Furthermore, job satisfaction was assessed using a single global item rather than a multidimensional validated instrument. Consequently, it was not possible to determine which specific organizational dimensions (e.g., leadership, staffing adequacy, remuneration, professional autonomy, career development opportunities, or collegial support) primarily contributed to respondents’ overall job satisfaction. The use of a single-item measure may also have introduced greater measurement error than multidimensional instruments and should therefore be considered when interpreting the magnitude of its association with burnout. Therefore, the strength of the observed association between job satisfaction and burnout should be interpreted with appropriate caution. Another limitation is that the survey did not assess individual recovery experiences following work, such as sleep quality, psychological detachment from work, leisure activities, physical activity, social support, or other recovery strategies. These factors may influence burnout levels and could partly explain individual variability beyond the organizational characteristics examined in the present study. Future research should integrate both organizational and recovery-related variables to provide a more comprehensive understanding of burnout among nurses. In addition, some occupational variables required harmonization from free-text responses, which may have introduced residual classification variability. Finally, although participants were recruited from all Italian macro-areas, the sample cannot be considered fully representative of the entire Italian nursing workforce. Moreover, the online survey format may have excluded nurses with limited digital access or lower engagement with professional social media platforms. Nonetheless, the relatively large sample size, geographical heterogeneity, and use of validated instruments provide meaningful and policy-relevant insights into burnout among Italian nurses [33,36,40]. Accordingly, the findings should be interpreted as exploratory but highly informative for organizational and occupational health research.

## 5. Conclusions

This nationwide online survey demonstrates that burnout among Italian nurses is widespread and strongly associated with organizational factors, particularly job satisfaction and workload. The strong inverse association between job satisfaction and burnout, together with the positive association observed for working more than 40 h per week, suggests that burnout cannot be effectively addressed through individual-level interventions alone [41,42].

The present findings, interpreted together with previous literature, support a systems-level approach to burnout prevention focusing on staffing adequacy, workload governance, leadership quality, and psychologically safe work environments. Contemporary evidence indicates that organizational interventions targeting these domains are associated with improved nurse retention, lower burnout levels, and better patient outcomes [21,27,32,35,36,37].

Given the established relationships between nurse burnout, patient safety, and workforce sustainability, healthcare systems should increasingly consider burnout indicators as relevant organizational performance and quality metrics alongside traditional clinical and financial indicators [29]. Integrating workforce well-being into governance frameworks, accreditation processes, and workforce planning should therefore be considered a strategic priority for healthcare organizations.

From a health systems perspective, preventing burnout should be considered a core organizational responsibility rather than solely an individual well-being initiative. Organizational policies aimed at improving working conditions, professional support, and workforce engagement have been associated in previous research with better workforce retention, greater organizational resilience, and improved patient safety outcomes. The present study provides additional observational evidence supporting these broader organizational hypotheses.

The present findings should be interpreted as identifying organizational priorities rather than prescribing specific interventions, which should be evaluated in future implementation studies.

Future longitudinal and multi-level research is needed to better clarify causal pathways and organizational determinants of burnout among nurses.

## Figures and Tables

**Table 1:** Sociodemographic and Professional Characteristics of the Sample (N = 557).

Characteristic	n	%
Gender		
Female	471	84.6
Male	85	15.3
Prefer not to say	1	0.2
Age group		
<30 years	206	37.0
30–39 years	191	34.3
40–49 years	76	13.6
≥50 years	84	15.1
Geographic area		
North	292	52.4
Centre	120	21.5
South	69	12.4
Islands	76	13.6
Educational level		
Bachelor’s degree	353	63.4
Postgraduate master	129	23.2
Master’s degree	48	8.6
Other	27	4.8
Years of experience		
<5 years	210	37.7
5–10 years	163	29.3
11–20 years	85	15.3
21–30 years	52	9.3
≥31 years	47	8.4
Contract type		
Permanent	470	84.4
Fixed-term	55	9.9
Freelance	22	3.9
Cooperative	9	1.6
Weekly working hours		
<30 h	19	3.4
30–36 h	253	45.4
37–40 h	246	44.2
>40 h	39	7.0
Job satisfaction		
1. Very low	54	9.7
2. Low	102	18.3
3. Moderate	212	38.1
4. High	154	27.6
5. Very high	35	6.3

Note. Percentages may not total 100 due to rounding. One participant had missing data for contract type.

**Table 2:** Descriptive Statistics of Copenhagen Burnout Inventory (CBI) Scores (0–100).

Measure	Mean ± SD	Median (IQR)	Min–Max
Personal burnout	60.11 ± 19.53	62.5 (45.8–75.0)	0–100
Work-related burnout	53.30 ± 22.25	53.6 (35.7–67.9)	0–100
Patient-related burnout	38.99 ± 22.77	37.5 (20.8–54.3)	0–100
Total CBI score	50.80 ± 18.87	51.2 (36.7–63.5)	3.8–100

**Table 3:** Burnout Severity Categories According to Total CBI Score.

Burnout Severity	CBI Total Score	n	%
Low burnout	<33	108	19.4
Moderate burnout	33–66	333	59.8
High burnout	>66	116	20.8

**Table 4:** Spearman Correlations Between Burnout Dimensions, Job Satisfaction, and Weekly Working Hours.

Variables	Spearman’s ρ
Personal vs. Work-related burnout	0.861 *
Personal vs. Patient-related burnout	0.521 *
Work-related vs. Patient-related burnout	0.569 *
Total burnout vs. Job satisfaction	−0.620 *
Total burnout vs. weekly working hours	0.180 *

* *p* < 0.001.

**Table 5:** Internal Consistency of the Copenhagen Burnout Inventory (CBI).

Scale	Cronbach’s Alpha
Personal burnout	0.878
Work-related burnout	0.904
Patient-related burnout	0.883
Total CBI scale	0.941

**Table 6:** Multivariable Linear Regression Analysis of Factors Associated with Total Burnout (CBI Total Score).

Predictor	β	SE	95% CI	*p*-Value	VIF
Job satisfaction	−11.16	0.60	−12.35 to −9.97	<0.001	1.04
Weekly hours > 40	7.05	2.45	2.23 to 11.86	0.004	1.02
Female gender	−0.50	1.76	−3.95 to 2.96	0.777	1.04
Age 30–39 years	0.52	1.98	−3.37 to 4.40	0.794	2.28
Age 40–49 years	−1.19	2.86	−6.81 to 4.43	0.678	2.50
Age ≥ 50 years	−12.83	4.24	−21.15 to −4.51	0.003	5.96
Experience 5–10 years	1.96	1.95	−1.87 to 5.79	0.315	2.04
Experience 11–20 years	0.93	2.61	−4.19 to 6.04	0.723	2.28
Experience 21–30 years	7.86	3.83	0.35 to 15.38	0.040	3.22
Experience ≥ 31 years	11.83	4.70	2.60 to 21.05	0.012	4.42
Geographic area: Centre	−0.13	1.60	−3.26 to 3.01	0.937	1.12
Geographic area: South	0.86	1.99	−3.05 to 4.77	0.666	1.11
Geographic area: Islands	−1.54	1.94	−5.34 to 2.27	0.428	1.15

Note. Reference categories: male gender, age < 30 years, professional experience < 5 years, and Northern Italy. Adjusted R^2^ = 0.398; model *p* < 0.001. VIF = Variance Inflation Factor. One participant who preferred not to disclose gender was excluded from the regression model; final model N = 556.

**Table 7:** Organizational Interpretation of the Present Findings and Evidence-Informed Management Implications.

Finding	Organizational Implication
High prevalence of moderate-to-high burnout	Need for routine organizational monitoring of burnout indicators
Strong association between job satisfaction and burnout	Importance of supportive leadership, organizational climate, and workforce engagement
Association between long working hours and burnout	Need for workload regulation and staffing adequacy strategies
Lower burnout among older nurses	Potential role of resilience, adaptation, and mentoring experience
Excellent internal consistency of the CBI	Suitability of the CBI as an organizational monitoring instrument

## Data Availability

The data presented in this study are available on request from the corresponding author due to privacy and ethical restrictions, as the dataset contains sensitive occupational health and sociodemographic information from a sample of healthcare professionals who were guaranteed anonymity.
